# The Bimodal Lifestyle of Intracellular *Salmonella* in Epithelial Cells: Replication in the Cytosol Obscures Defects in Vacuolar Replication

**DOI:** 10.1371/journal.pone.0038732

**Published:** 2012-06-13

**Authors:** Preeti Malik-Kale, Seth Winfree, Olivia Steele-Mortimer

**Affiliations:** *Salmonella* Host-Cell Interactions Section, Laboratory of Intracellular Parasites, Rocky Mountain Laboratories, National Institute of Allergy and Infectious Disease, National Institutes of Health, Hamilton, Montana, United States of America; University of Louisville, United States of America

## Abstract

*Salmonella enterica* serovar Typhimurium invades and proliferates within epithelial cells. Intracellular bacteria replicate within a membrane bound vacuole known as the *Salmonella* containing vacuole. However, this bacterium can also replicate efficiently in the cytosol of epithelial cells and net intracellular growth is a product of both vacuolar and cytosolic replication. Here we have used semi-quantitative single-cell analyses to investigate the contribution of each of these replicative niches to intracellular proliferation in cultured epithelial cells. We show that cytosolic replication can account for the majority of net replication even though it occurs in less than 20% of infected cells. Consequently, assays for net growth in a population of infected cells, for example by recovery of colony forming units, are not good indicators of vacuolar proliferation. We also show that the *Salmonella* Type III Secretion System 2, which is required for SCV biogenesis, is not required for cytosolic replication. Altogether this study illustrates the value of single cell analyses when studying intracellular pathogens.

## Introduction

*Salmonella enterica* serovar Typhimurium (*Salmonella* Typhimurium) is a facultative intracellular pathogen, which is a common cause of gastroenteritis in humans. The ability of *Salmonella* to establish its intracellular niche is dependent on two Type Three Secretion Systems (T3SS). T3SS1, encoded by *Salmonella* Pathogenicity Island (SPI) 1, is required for efficient invasion of nonphagocytic cells. In contrast, the SPI2-encoded T3SS2 is induced following internalization of *Salmonella* into host cells and is required for post-invasion processes. Together T3SS1 and T3SS2 translocate over 30 effector proteins into the host cell where they interact with a variety of targets [1].

In epithelial cells *Salmonella* Typhimurium has a bimodal lifestyle, replicating in a membrane bound compartment known as the *Salmonella* containing vacuole (SCV) [2] as well as in the cytosol [3], [4], [5], [6], [7]. The SCV is a modified phagosome, characterized by the presence of lysosomal membrane proteins, low pH and sustained dynamic interactions with the endocytic and biosynthetic pathways [8], [9], [10], [11], [12], [13]. Early maturation of the SCV involves acquisition of lysosomal membrane proteins, such as lysosomal-associated membrane protein-1 (LAMP1) within 1–2 h following invasion [14], and movement from the cell periphery to a juxtanuclear location [15], [16]. Bacterial replication is initiated following LAMP1 acquisition [14], [17], [18], [19] and is accompanied by the appearance of dynamic membrane tubules that extend from the surface of the SCV [20], [21]. Effectors translocated by the SPI2-encoded T3SS2, are essential for maturation of the replicative SCV. Bacteria lacking a functional T3SS2, and therefore unable to translocate any effectors, remain within an immature SCV, which is LAMP1-positive but does not form membrane tubules and is defective at juxtanuclear positioning [15]. These mutants also have an intracellular replication defect, in both macrophages and epithelial cells, although in epithelial cells the defect is not apparent during the initial replication phase [3], [4], [5], [6], [7], [20], [22], [23], [24], [25].

While the SCV has been extensively studied, little is known about how *Salmonella* adapt to and/or modify the cytosolic niche. Using a polarized epithelial cell model Knodler *et al* showed that cytosolic *Salmonella* Typhimurium replicate to higher numbers than vacuolar bacteria, a phenotype dubbed “hyper-replication" [6]. This study also showed that these two intracellular populations of bacteria are transcriptionally distinct: the intravacuolar bacteria are SPI2-induced whereas the cytosolic bacteria are SPI1-induced and flagellated. Epithelial cells containing hyper-replicating SPI1-induced *Salmonella* undergo inflammatory cell death, marked by loss of plasma membrane integrity and activation of caspase 1 and caspase 3/7. Ultimately these cells are extruded from monolayers, both *in vivo* and *in vitro*, and the invasion-primed *Salmonella* are released into the extracellular milieu [6].

Here we have investigated whether cytosolic replication of *Salmonella* contributes significantly to net growth in HeLa cells, which are commonly used to study *Salmonella*-host cell interactions *in vitro*. In addition, we have compared the requirement for T3SS2 in cytosolic *vs* vacuolar replication. Since cytosolic *Salmonella* are SP11-induced, and do not express detectable levels of SPI2 genes [6], it seems probable that SPI2 is not required for hyper-replication, although this has not been directly demonstrated. If T3SS2 is not required for cytosolic replication this could explain why bacteria lacking T3SS2 have a delayed replication defect in epithelial cells [3], [4], [6], [7], [22], [26], since cytosolic replication could potentially obscure defects in vacuolar replication. We used microscopy-based approaches, in both fixed and living cells, to assess the replication over time of both the vacuolar and cytosolic populations of *Salmonella* in individual epithelial cells. Our results show that, although cytosolic *Salmonella* Typhimurium occur in a minority of infected epithelial cells, the hyper-replication of these bacteria accounts for a significant proportion of net bacterial replication. Furthermore, cytosolic replication is SPI2-independent and can obscure replication defects in vacuolar bacteria.

## Results

### Analysis of Intracellular Replication of *Salmonella*

**Figure 1:**
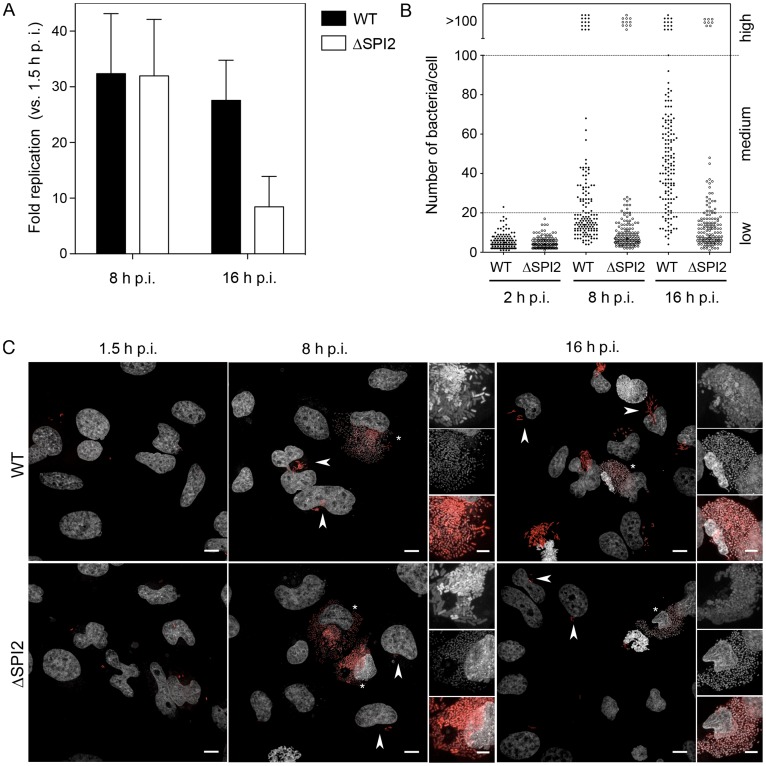
Analysis of heterogeneity of intracellular *Salmonella* replication. (A) Gentamicin protection assay. HeLa cells infected with either WT or mutant (ΔSPI2) bacteria were harvested at 1.5, 8 and 16 h p.i. The fold change in recoverable colony forming units was calculated versus 1.5 h p.i. Shown are the means ± SD. (B) Intracellular mCherry *Salmonella*. For each time point WT and mutant bacteria were counted in ≥50 HeLa cells in each of 3 independent experiments. Shown are the combined data. (C) Representative images of infected HeLa cell monolayers used for analysis in (B). DNA was stained with DAPI. Images are maximum z-projections of 0.32 µm sections. Insets are magnified regions with the red channel (mCherry) gamma adjusted (0.6) to show both high and low fluorescent intensity *Salmonella*. Arrows indicate low or medium replication phenotypes and asterisks indicate high replication phenotype. Scale bars are 10 µm (5 µm in insets).

Following internalization into epithelial cells *Salmonella* Typhimurium replicates within the SCV but also in the cytosol [3], [4], [5], [6], [7]. However, the relative contribution of the two distinct intracellular populations to net replication remains undefined. To address this question we analyzed intracellular replication in cultured epithelial cells by both the standard gentamicin protection assay, which measures net replication, and a microscopy-based technique to follow bacterial replication in single cells. Many studies have shown robust replication of wild type (WT) *Salmonella* Typhimurium in epithelial cells (∼20–30 fold over 8 h of infection, Fig. 1A) [3], [5], [15], [20], [23], [25], [27], [28]. However, although the SPI2-encoded T3SS2 is required for vacuole biogenesis, we saw no defect in net replication for a SPI2 deletion mutant (ΔSPI2) over this time period. In contrast, at 16 h post infection (p.i.), there was a significant reduction in the amount of recoverable intracellular bacteria for the SPI2 mutant. We next used standard fluorescence microscopy to examine the numbers of bacteria in individual cells at these times. For ease of detection, particularly in the subsequent live cell experiments, we used bacteria constitutively expressing the fluorescent protein mCherry (mCherry *Salmonella*). HeLa cells infected with mCherry *Salmonella* were fixed at 2, 8 and 16 h p.i. and intracellular bacteria then enumerated by fluorescence microscopy (Fig. 1B and 1C). As expected, since the SPI2-encoded T3SS2 is not required for invasion, no difference was seen in the numbers of WT or SPI2 mutant intracellular bacteria at the earliest time point (2 h p.i.). The mean number of bacteria per infected cell was 5±4 and 5±3 (mean ± SD) for the WT and mutant respectively. In contrast, at 8 h p.i., following the onset of replication, the numbers of bacteria per cell varied dramatically. In some cells the bacteria occupied the entire cytoplasm of the cell and could not be accurately enumerated (TNTC, >100 bacteria), whereas at the other extreme many cells contained less than 10 bacteria. To facilitate analysis, we separated cells into three groups based on the numbers of intracellular bacteria; low (1–20 bacteria), moderate (20–100 bacteria) and high (>100 bacteria) (Fig. 1B). We then compared the frequency of each phenotype in cells infected with either WT *Salmonella* or the SPI2 mutant. At 8 h p.i. cells falling into the high (>100 bacteria) group accounted for 10±4 (mean ± SD) % of WT infected cells compared to 7±1% of the SPI2 mutant infected cells. In comparison, the moderate (20–100 bacteria per cells) group was more frequent in WT infected cells (29±17%) compared to those infected with the SPI2 mutant (6±6%). At 16 h this effect was enhanced with 75±5% of WT infected cells containing 20–100 bacteria per cell compared to 14±5% of SPI2 mutant infected cells. In contrast, cells containing >100 bacteria made up 8±5% or 5±3% of the population for WT infected and SPI2 mutant infected cells respectively. These results indicate that the SPI2-encoded T3SS2 is required for development of the subpopulation of cells that contain 20–100 bacteria but not the subpopulation that contains >100 bacteria.

### Live Cell Imaging Defines a Role for SPI2 Early in Infection

**Figure 2:**
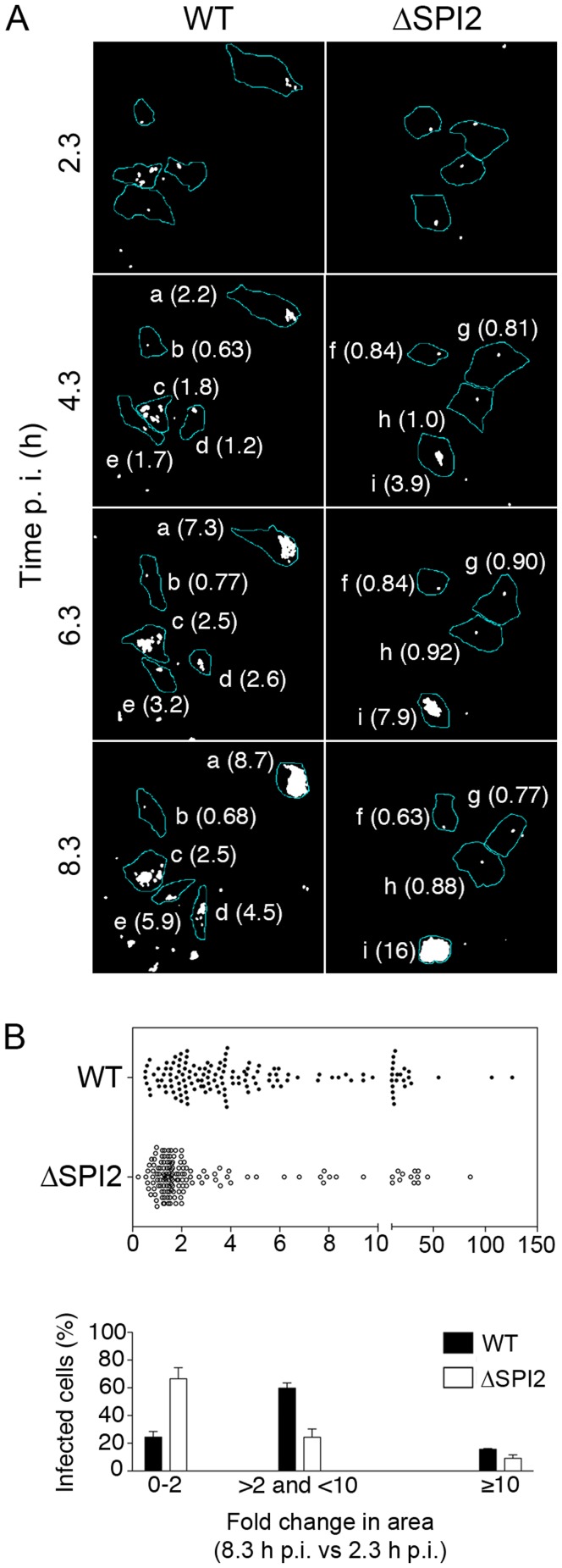
SPI2-independent replication in HeLa cells. (A) Time lapse microscopy of HeLa cells infected with mCherry *Salmonella*. Numbers on the left indicate the time p.i. Individual host cells are outlined (a–e, WT infected cells; f-i, ΔSPI2 infected cells). For each cell the net change in area versus 2 h 20 min p.i. (ΔArea) occupied by mCherry *Salmonella* is indicated in parentheses. (B) Graphical representation of data shown in (A) for showing fold increase at 8 h 20 min. Data represents at least five fields from two wells in each of 3 independent experiments. Shown are the combined data. In lower panel the same data is shown in a histogram. Cells were divided into three categories depending on the ΔArea. Shown are the means ± SD of a total of 140 HeLa cells each for WT and ΔSPI2 (n = 3).

To further investigate the role of SPI2 in infected HeLa cells we used a live cell imaging analysis method in which the increase in area occupied by mCherry fluorescent *Salmonella* over time approximates bacterial replication [29]. Infected HeLa cells were imaged from ∼2 to 8 h p.i. (Fig. 2A, Movies S1 and S2) with images taken at 10 min intervals. Post acquisition analysis was then carried out to estimate the net change in area occupied by mCherry *Salmonella*, obtained by dividing the pixel area occupied by intracellular bacteria at each time point by the area occupied at ∼2 h p.i. (ΔArea; see Materials and Methods section for details). The data are shown as a scatter plot in which data points have been binned into three groups representing low (ΔArea <2), moderate (ΔArea >2−<10) and high (ΔArea ≥10) replication (Fig. 2B). After 8 hours of infection, the percentage of cells containing large numbers of bacteria was slightly higher for WT (16±0%, mean ± SD) compared to SPI2 mutant infected cells (9±1%) although this difference was not seen in the previous experiment using fixed cells (Fig. 1B and C). However, whereas the majority of cells infected with WT bacteria fell into the moderate replication group, 60±2% the majority of cells infected with the SPI2 mutant fell into the low replication group, 66±5%. Thus, with the noted exception at 8 h p.i., these live-cell data are consistent with the data shown in Figure 1, and confirm a requirement for SPI2 in replication of a subpopulation of bacteria i.e. those falling in the moderate replication group.

### Cytosolic Replication in HeLa Cells is SPI2-independent

**Figure 3:**
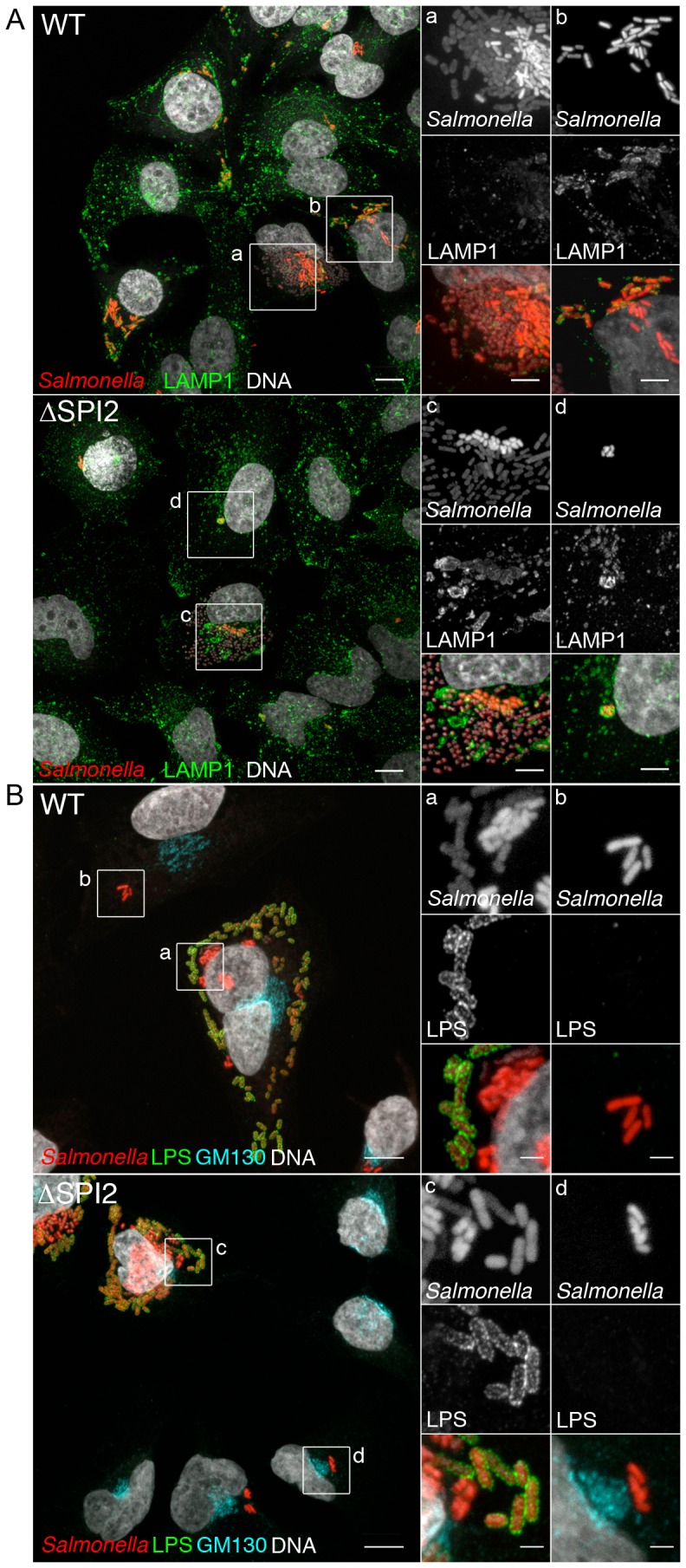
SPI2-independent replication occurs in the cytosol. (A) Replicating SPI2 mutant *Salmonella* are LAMP1 negative. HeLa cells infected with mCherry *Salmonella* (red) were fixed at 8 h p.i. and stained for the SCV marker LAMP1 (green) and DNA (gray). (B) Replicating SPI2 mutant *Salmonella* are in the cytosol of HeLa cells. HeLa cells were infected with mCherry *Salmonella*, either WT or ΔSPI2, fixed at 6 h p.i. and processed for digitonin-selective permeabilization. Insets *a* and *c* include cytosolic hyper-replicating bacteria and insets *b* and *d* include putative vacuolar bacteria. Images are maximum z-projections of 0.32 µm sections. Insets are magnified regions with the red channel (mCherry) gamma adjusted (0.6) to show both high and low fluorescent intensity *Salmonella*. Scale bars are 10 µm (2 µm in insets).

We next carried out experiments to determine the intracellular location of replicating *Salmonella*. To differentiate between vacuolar and cytosolic bacteria HeLa cells were stained for the lysosomal transmembrane protein LAMP1, which accumulates on the membrane of SCVs. At 8 h p.i. most infected cells contained mCherry *Salmonella* enclosed within LAMP1-positive vacuoles (Fig. 3A) although in cells containing large numbers of *Salmonella* (>100) the majority of bacteria were LAMP1-negative. To confirm that the LAMP1-negative *Salmonella* were cytosolic we used digitonin permeabilization to selectively permeabilize the plasma membrane without affecting the integrity of intracellular membranes including that of the SCV. This selective permeabilization provides access for anti-LPS antibodies to label cytosolic but not vacuolar *Salmonella*. At 6 h p.i. ∼15% of infected cells contained mCherry *Salmonella* that stained with anti-LPS antibody (Fig. 3B). No difference could be detected in the frequency of cytosolic WT and SPI2 mutant bacteria in HeLa cells (Fig. 3, and data not shown), indicating that cytosolic replication is SPI2-independent.

### Replication in the SCV, but Not in the Cytosol, is SPI2 Dependent

**Figure 4:**
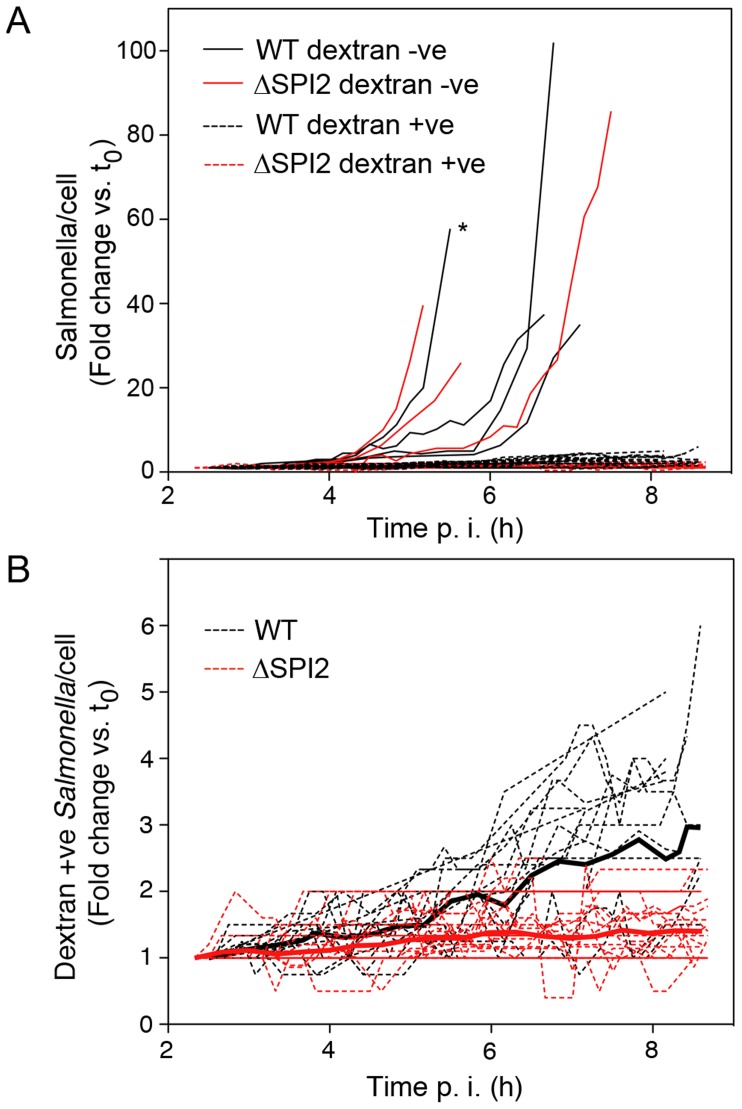
Quantification of intracellular replication. To differentiate between vacuolar and cytosolic *Salmonella*, internalized fluorescent dextran was used as a marker of intact SCVs. 488-dextran was internalized into HeLa cells overnight before infection with mCherry *Salmonella.* Live cell imaging was initiated at 2–3 h p.i. At 10 min intervals nine overlapping fields were collected, centered on a total of 5–10 infected cells for each experiment. Subsequently the fields were stitched together and the dextran-negative (cytosolic) and dextran-positive (vacuolar) bacteria enumerated. In total 47 bacterial microcolonies were assayed, 7 dextran-negative and 40 dextran-positive, from 8 independent experiments. (A) Replication of both cytosolic (solid lines) and vacuolar bacteria (dashed lines). * cell lifted off dish, † TNTC. (B) Enlarged graph to show replication of vacuolar bacteria.

To confirm that cytosolic replication is SPI2 independent we adapted the live-cell imaging approach to compare the rates of bacterial replication in the cytosol and the SCV. To differentiate between vacuolar and cytosolic bacteria, we used fluorescent dextran, a fluid phase marker that efficiently labels the endocytic pathway and accumulates within the *Salmonella* containing vacuole (SCV) [13]. Alexa488-Dextran was internalized into the Hela cells overnight and the cells then infected with mCherry *Salmonella*. To increase the number of infected cells, which could be simultaneously imaged in each experiment, nine overlapping fields were imaged and later stitched together for image analysis. In this way we imaged a total of 47 infected cells; 19 WT-infected and 28 SPI2 mutant-infected (Fig. 4). At the initiation of imaging (2–3 h p.i.) the number of bacteria per infected cell was 3±1 (mean ± SD) for WT and 5±1 for the mutant. Dextran-negative (cytosolic) bacteria were observed in 4 out of 19 WT infected cells compared to 3 out of 28 mutant infected cells. Cytosolic replication was first observed between 4 and 6 h p.i. for both WT and mutant bacteria and resulted in 20–100 fold increases in bacterial numbers, beyond which increases could not be accurately measured (Fig. 4A and Movies S3, S4, S5, S6, compare vacuolar bacteria shown in Movie S3 and S4 with cytosolic bacteria shown in Movie S5 and S6). One WT-infected cell in this group detached from the dish during imaging after an ∼6 fold increase in intracellular bacteria. Replication of dextran positive (vacuolar) bacteria, which was also initiated by 4–5 h p.i., was only apparent when plotted on a different scale (Fig. 4B). By 9 h p.i. WT vacuolar bacteria increased by ∼3 fold with the largest observed increase in a single cell being 6 fold. In comparison, the vacuolar SPI2 mutant bacteria increased by only ∼1.4 fold and the largest observed increase was 2.3 fold. These live cell experiments confirm that SPI2 is required for *Salmonella* replication in the vacuole but not for cytosolic replication.

### Low Magnification Live Cell Imaging of *Salmonella*-infected HeLa Cells Provides Novel Insight into Intracellular Populations

**Figure 5:**
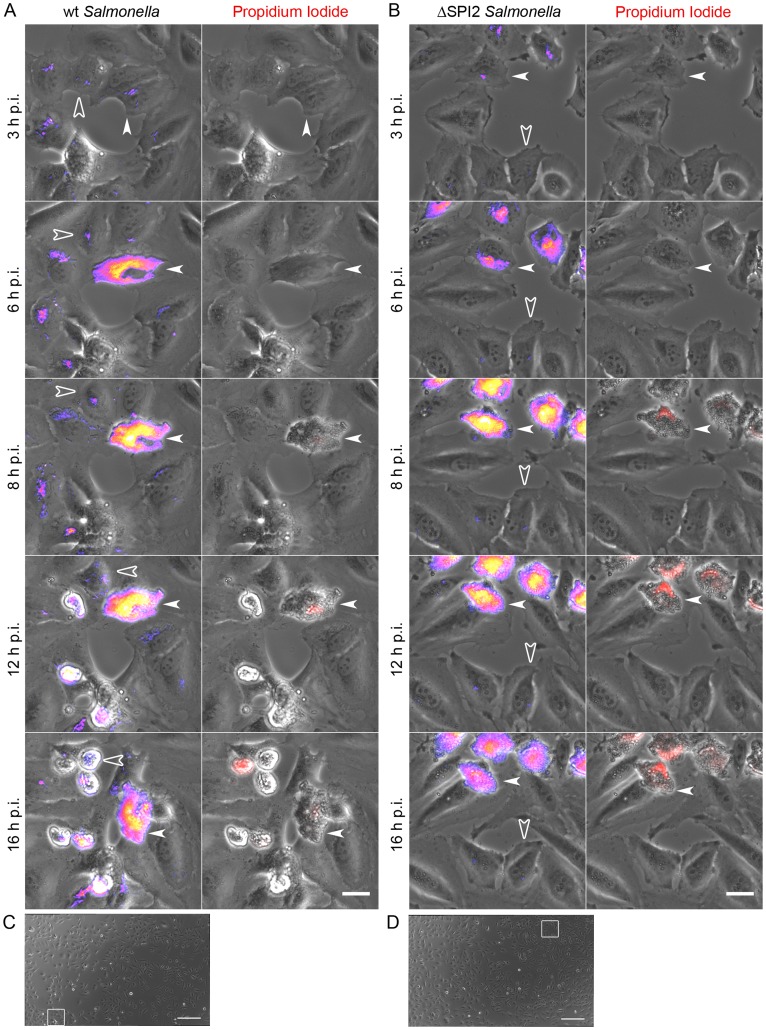
Contribution of hyper-replicating *Salmonella* to net replication. HeLa cell monolayers were infected with GFP *Salmonella*. Propidium iodide was added at 1.5 h p.i. to identify cells with compromised plasma membranes. Live cell imaging was initiated at 2–3 h p.i. At 20 min intervals 20 overlapping fields were collected, for ∼200 infected cells for each experiment. Subsequently the fields with stitched together. (A, B) Magnified regions from stitched fields as indicated in C and D for WT and SPI2 mutant infections respectively. Panels on the left show an overlay of GFP-*Salmonella* artificially colored to reveal both the lowest (blue/purple) and highest (yellow/white) intensity bacteria and phase-contrast (gray). Panels on the right show an overlay of propidium iodide staining (red) and phase-contrast (gray). Arrowheads indicate hyper-replicating bacteria and open arrowheads indicate bacteria not hyper-replicating. Scale bars are 25 µm. (C, D) Stitched fields for WT and SPI2 mutant infection with regions for A and B marked with white squares. Scale bars are 250 µm.

A major limitation of live cell imaging experiments, such as those described above is that only a few cells can be imaged at one time. To overcome this limitation we refined the multiple-field live cell imaging approach, using low magnification (20X objective) and imaging 20 overlapping fields, to allow simultaneous imaging of ∼500 cells. For these experiments we used *Salmonella* constitutively expressing GFP (GFP *Salmonella*) and propidium iodide was added to the tissue culture media following infection, to allow identification of cells with compromised plasma membrane. Imaging was initiated 2.5–3 h p.i. and images were then acquired at 20 min intervals until 18 h p.i. Representative time-lapse movies (Movies S7 and S8) from one of three independent experiments reveal ongoing cell division as well as cells rounding up and detaching, with the later being particularly apparent at the later time points. GFP *Salmonella* can be observed in many HeLa cells, although at early time points the infected cells are not always easy to identify since there are few bacteria per cell. In contrast, cells containing hyper-replicating bacteria are readily identified from 4 h p.i. and by 8 h p.i. 11±3% (mean ± SD, n = 3) of WT-infected and 17±3% of SPI2 mutant-infected cells fell into this category (Movies S7 and S8 respectively, and still images from the movies shown in Fig. 5C and 5D with selected regions in Fig. 5A and 5B). Although some of these cells were observed detaching from the monolayer, the percentage of infected cells containing hyper-replicating bacteria did not change significantly between 8 and 16 h for either WT-infected or SPI2 mutant-infected cells.

As previously reported epithelial cells containing hyper-replicating bacteria undergo inflammatory death, characterized by activation of caspases 1 and 3/7 as well as loss of plasma membrane integrity [6]. Here we also observed that from 8 h p.i. cells containing hyper-replicating bacteria also stained with PI, indicating loss of membrane integrity (Fig. 5 and Movies S7 and S8). Although the cells containing hyper-replicating bacteria are very conspicuous in these live cell experiments, the majority of infected cells (80–90%) contain bacteria undergoing limited replication. Very few of these cells stained with PI during the course of the experiment indicating that the plasma membrane is intact and the infected cells remain viable. Intriguingly, some of these cells do round up and detach from the monolayer although the significance of this is unclear. Comparison of movies S7 and S8, which show WT- and SPI2-mutant-infected cells respectively, reveals that intermediate replication occurs frequently in WT-infected cells but not in cells infected with the mutant (See also Fig. 5). Since this reproduces the data shown in Fig. 1 and Fig. 2 we have not quantified these populations in these experiments. These low-magnification live cell experiments demonstrate conclusively that the SPI2 encoded T3SS is not required for cytosolic replication of *Salmonella.* In addition, they reveal the potential for hyper-replicating bacteria to equal or even outnumber the vacuolar population in infected HeLa cells.

## Discussion

Historically the SCV has been considered the primary site of replication for intracellular *Salmonella*. However, it is now clear that this facultative intracellular pathogen has a distinct bimodal lifestyle in epithelial cells [6]. Here we have analyzed the contributions of vacuolar and cytosolic replication to net intracellular growth. Our data shows that, although the majority of infected HeLa cells contain vacuolar *Salmonella* these bacteria replicate rather inefficiently, ∼3 fold over 8 h, and can not account for the net levels of intracellular replication in these cells. In contrast, while less than 20% of infected cells contained cytosolic bacteria this population replicated 40 fold or more so that the epithelial cells were rapidly filled with bacteria. Thus, in epithelial cells, net replication of intracellular *Salmonella* is a reflection of both cytosolic and vacuolar replication.

Although the requirement for the SPI2-encoded T3SS2 in macrophages has been unequivocally demonstrated [30], [31], [32], [33], the role of this important virulence determinant in epithelial cells has been less well established [3], [5], [22], [28]. While many studies have shown that SCV biogenesis is dependent on the T3SS2, mutants lacking the ability to translocate any T3SS2 effectors often show little or no defect in replication during the first 8 h following invasion when compared to 16 h p.i. where they are significantly defective in replication [3], [5]. But those experiments primarily used gentamicin protection to quantify intracellular bacteria. Our results show that a defect in vacuolar replication is likely obscured by T3SS2-independent cytosolic replication when net replication is assayed at earlier time points during infection (i.e. 8 h). Then why is the growth defect apparent at later time points? One possibility is that cells containing hyper-replicating bacteria more easily detach from the monolayer. In this case a standard gentamicin-assay, in which monolayers of infected cells are solubilized in detergent, so that recoverable cfus can be estimated by plating, would underestimate hyper-replicating bacteria. This may well be an issue for the gentamicin assay, or other protocols that involve rinsing of monolayers. However, our live cell experiments involve no rinsing steps after the change to low gentamincin at 1.5 h and under these conditions very few cells containing hyper-replicating bacteria were seen detaching from the surface. Instead, we believe that loss of plasma membrane integrity in cells containing hyper-replicating bacteria, as shown here and previously [6], allows entry of gentamicin resulting in killing of hyper-replicating bacteria. Since PI staining in these cells is not apparent until after 8 h p.i., by which time cytosolic replication has already exceeded vacuolar replication, we propose that gentamicin killing of this population will not occur until later time-points. Selective removal of hyper-replicating bacteria, by loss of cells from the monolayer and/or by gentamicin killing, would explain why the SPI2-mutant, which is defective only in vacuolar replication, does not show a replication defect until later time points in gentamicin-protection assays. Both vacuolar and cytosolic populations will contribute to the net replication observed at 8 h p.i. whereas at later time points, such as16 h p.i., the cytosolic population will underestimated because of killing by gentamicin and loss of cells (see Fig. 1). This has very significant ramifications when gentamicin assays are used to assess growth of *Salmonella* in epithelial cells, but not in macrophages where cytosolic *Salmonella* are unable to survive or grow [3], [4], [5], [6], [7], [20], [22], [23], [24], [25].

Transcriptome analysis of *Salmonella* Typhimurium in epithelial cells suggested that the SPI1 and SPI2 regulons together with flagellar genes are simultaneously expressed in vacuolar bacteria [34]. Instead we propose that this can be explained by the presence of two transcriptionally distinct populations of intracellular *Salmonella*, the vacuolar SPI2-induced population and the cytosolic SPI1-induced and flagellated population [6], that we have shown are both present in significant numbers. Further work is required to determine other differences in the expression profiles of these distinct populations of bacteria and to identify the bacterial factors involved in determining intracellular localization.

In conclusion, we report here that cytosolic SPI2-independent replication of *Salmonella* Typhimurium contributes significantly to net replication in epithelial cells. This has important implications for studies involving epithelial cells, however, further investigations are required to dissect the roles of cytosolic versus vacuolar replication in pathogenesis of this important bacterial pathogen.

## Materials and Methods

### Cell Culture and Bacterial Strains

The *Salmonella enterica* serovar Typhimurium strains used in this study, SL1344 wild type (WT) and ΔSPI2::Kan have been described previously [8]. SL1344 WT harboring pFPVmcherry or pFPV-25.1 for constitutive production of mCherry (mCherry *Salmonella*) or GFP (GFP *Salmonella*) have also been described [35]. The SPI2 mutant GFP *Salmonella* was generated by transforming SL1344 ΔSPI2::Kan with pFPV-25.1. For routine propagation, bacteria were grown on Luria-Bertani (LB) agar plates and stored at 4°C for up to one week. HeLa (human cervical adenocarcinoma, ATCC CCL-2) cells were used prior to passage number 14 following receipt from ATCC. Cells were grown in Growth Media (GM), consisting of Eagle’s minimal essential medium (MediaTech) supplemented with 10% (vol/vol) fetal bovine serum (Invitrogen), at 37°C in 5% CO_2_.

### Infection of Cultured Epithelial Cells with *Salmonella*

HeLa cells were seeded on plastic (Costar) or glass-bottom (Sensoplate; Grenier Bio-One) 24 well tissue culture plates or glass coverslips, 18–24 h before infections. Cells were infected with SPI1-induced bacteria as described previously [27]. Briefly, cultures grown overnight in LB-Miller broth with shaking (250 rpm) at 37°C were subcultured (1∶33) in 10 ml of fresh LB-Miller and grown shaking at 37°C to late log phase (3.5 h). *Salmonella* were then collected by centrifugation and resuspended in Hank’s buffered saline solution. HeLa cells were immediately infected at an MOI of ∼50∶1 for 10 minutes at 37°C, followed by three brief rinses in PBS and incubated in GM for 20 min at 37°C. Cells were then incubated in GM containing 50 µg/ml gentamicin for 1 h, to kill extracellular bacteria, followed by GM containing 10 µg/ml gentamicin for the remainder of the infection. To determine the number of viable intracellular bacteria, HeLa cells were lysed in 0.2% sodium deoxycholate in PBS at 1.5 and 8 h post infection and 10 fold serial dilutions plated on LB agar. Serial plating was also used to estimate the number of bacteria in the innoculum.

### Immunofluorescence Staining

HeLa cells were fixed in 2.5% paraformaldehyde for 10 min at 37°C. All subsequent steps were carried out at room temperature. Cells were blocked and permeabilized in PBS containing 10% (v/v) normal goat serum (NGS) and 0.1% (w/v) saponin (SS-PBS) for 10 min. They were then incubated with mouse monoclonal anti-LAMP-1 antibody (1∶1,000, clone H4A3, Developmental Studies Hybridoma Bank) for 45–60 min in SS-PBS, washed 3 times in PBS, 0.1% (w/v) saponin, incubated with Alexa Fluor 488-conjugated goat anti-mouse IgG (1∶800, Life Technologies) in SS-PBS for 45 min. Finally the cells were washed and mounted on glass slides using ProLong Gold antifade reagent containing the nuclear counterstain DAPI (Life Technologies).

### Differential Digitonin Permeabilization Assay

To determine whether intracellular bacteria were vacuolar or cytosolic we used a differential digitonin permeabilization assay [6]. Briefly, HeLa cells were infected with mCherry *Salmonella* (WT or ΔSPI2 mutant). At 6 h p.i. cells were washed three times in KHM buffer [110 mM potassium acetate, 20 mM Hepes, 2 mM MgCl_2_ (pH 7.3)], incubated with 150 µg/mL digitonin (Sigma) in KHM buffer for 1 minute, then immediately washed twice with KHM buffer. This and all subsequent steps were carried out at room temperature unless noted. To label cytosolic bacteria and the cytosolic face of the Golgi cells were then incubated in rabbit polyclonal anti-*Salmonella* lipopolysaccharide (LPS) antibody (1∶200; Difco) and mouse anti-human GM130 monoclonal antibody (1∶200; BD Transduction Laboratories) for 12 min at 37°C. Cells were washed twice with PBS and then fixed in 2.5% paraformaldehyde for 10 min at 37°C. Fixed cells were permeabilized in SS-PBS for 15 min followed by incubation with Alexa Fluor 488-conjugated goat anti-rabbit IgG and Alexa Fluor 647-conjugated goat anti-mouse IgG at 1∶400 for 45 mins. For each experiment, two coverslips were used as a permeabilization control to ensure that the plasma membrane, but not endomembranes, was permeabilized. For this, digitonin-treated cells were incubated with rabbit polyclonal antibody directed against the cytoplasmic tail of calnexin (1∶200; Stressgen, Enzo Life Sciences) and a mouse monoclonal antibody directed against rat luminal protein disulphide isomerase (1∶200; Thermo Scientific). Finally, coverslips were washed in PBS and mounted on glass slides as above.

### Fixed Cell Imaging

Cells were visualized and enumerated using inverted Nikon microscopes, Eclipse TE2000 or Ti-E, fitted with a Plan Apo 60X 1.4 NA oil immersion lens and B-2E/C or Y-2E/C filter blocks (Nikon Instruments). To determine the number of bacteria per cell, 150 infected cells were enumerated at each time point.

Confocal images were acquired on an LSM 710 controlled by the software package ZEN (Zeiss) fitted with a Plan-Apochromat 63X 1.40 NA oil immersion lens. Pixel sizes were 0.75–0.77 µm and z-steps for optical sections were 32 µm. Excitation and band-pass cut offs were as follows: DAPI, excitation 405 nm, emission 410–495 nm; Alexa Fluor 488, excitation 488 nm, emission 493–581 nm; mCherry, excitation 561 nm, emission 578–696 nm.

### Live Cell Imaging

HeLa cells grown on glass-bottom 24 well plates were infected with mCherry *Salmonella* or GFP *Salmonella*. When Dextran Alexa Fluor 488 (10,000 MW, Molecular Probes/Invitrogen) was used to identify vacuolar bacteria it was internalized overnight (0.2 mg/ml) prior to infection [13]. To stain HeLa cells with compromised plasma membranes, propidium iodide (1 µg/ml) was added 1.5 h p.i and maintained for the remainder of the infection. Live cell imaging experiments were performed using a spinning disc confocal system [6] consisting of a CSU10 spinning disk confocal (Yokogawa) with a custom AOTF-shuttered diode laser launch for excitation (Praire Technologies), fitted to a Ti-E inverted microscope with Perfect Focus System (Nikon Instruments) controlled by Metamorph v 7.7.0 (Molecular Devices) or by widefield with the same instrument fitted with an HQ_2_ interline CCD camera (Photometrics) and excitation with an EXFO metal halide light source (Lumen Dynamics). Cells were maintained at 37°C with 5% CO_2_ throughout imaging using a stage top incubation system (Pathology Devices). Excitation and filters were as follows: Alexa Fluor 488, excitation 488 nm, emission 525/50 nm; mCherry, excitation 561 nm, emission 600/45 nm. Filter blocks and lightpath for widefield imaging were as follows: GFP, B-2E/C; propidium iodide (PI), Y-2E/C; and phase contrast with phase plate Ph1 with a centered NCB filtered halogen light source focused with an ELWD condenser configured for Köhler illumination (Nikon Instruments).

For low magnification imaging of *Salmonella* replication, using a Plan Fluor 20X 0.5 NA ELWD non-immersion phase contrast objective lens (Nikon Instruments), nine fields, selected systematically in a non-overlapping 3×3 grid, were imaged sequentially (single optical sections) for Alexa Fluor 488 and mCherry at 10 min intervals. Alternatively, twenty overlapping fields selected systematically were imaged sequentially (widefield) for GFP, PI and phase contrast at 20 min intervals.

All post-acquisition analysis was carried out using ImageJ software (W.S. Rasbans, National Institutes of Health, Bethesda, MD). To measure the area occupied by *Salmonella* within individual HeLa cells, a region-of-interest was selected by thresholding on the red channel so as to include all fluorescent mCherry *Salmonella*. To determine the fold-change in area (ΔArea) for each infected HeLa cell, the area occupied by *Salmonella* at 8 h 20 min p.i. was normalized to the area occupied by *Salmonella* at 2 h 20 min h p.i. Overlapping fields were stitched together using the Stitch Grid of Images plugin from the ImageJ derivative, Fiji v1.43-1.46a [36].

For high magnification imaging of *Salmonella* replication, using a Plan Apo 60X 1.4 NA oil immersion lens (Nikon Instruments) fitted with a temperature regulated heating collar (Bioptechs), nine overlapping fields (total of 5–10 infected cells) were imaged sequentially (single optical sections) for Alexa Fluor 488 and mCherry at 10 min intervals. Overlapping fields were stitched together as given above. Individual *Salmonella* were enumerated for each infected cell at each time point time and normalized to the number at the initial time-point.

### Statistics

Statistical tests, including unpaired Student’s t-test for Gaussian distributions, Mann-Whitney for non-parametric distributions and ANOVA and 2-way ANOVA for multiple category Gaussian distributions, were performed using Prism (Graphpad).

## Supporting Information

Movie S1 **Live-cell imaging of** ***Salmonella*** **Typhimurium WT replication in HeLa cells at 20X magnification. Internalized 488-dextran was used to identify vacuolar** ***Salmonella.*** Left panel shows mCherry *Salmonella* artificially colored to reveal both low (blue/purple) and high (yellow/white) intensity bacteria. Right panels show an overlay of mCherry *Salmonella* (red) and Dextran-488 (green). Compare dextran positive vacuolar bacteria to dextran negative cytosolic bacteria. Times post infection are indicated.
(MOV)

Movie S2 **Live-cell imaging of** ***Salmonella*** **Typhimurium SPI2 mutant replication in HeLa cells at 20X magnification. Internalized 488-dextran was used to identify vacuolar** ***Salmonella.*** Left panel shows mCherry *Salmonella* artificially colored to reveal both low (blue/purple) and high (yellow/white) intensity bacteria. Right panels show an overlay of mCherry *Salmonella* (red) and Dextran-488 (green). Compare dextran positive vacuolar bacteria to dextran negative cytosolic bacteria. Times post infection are indicated.
(MOV)

Movie S3 **Live-cell imaging of** ***Salmonella*** **Typhimurium WT replication in HeLa cells at 60X magnification, showing vacuolar replication.** Internalized 488-dextran was used to identify vacuolar *Salmonella.* Left panel shows mCherry *Salmonella* artificially colored to reveal both low (blue/purple) and high (yellow/white) intensity bacteria. Right panels show an overlay of mCherry *Salmonella* (red) and Dextran-488 (green). Compare dextran positive vacuolar bacteria to dextran negative cytosolic bacteria. Times post infection are indicated.
(MOV)

Movie S4 **Live-cell imaging of** ***Salmonella*** **Typhimurium SPI2 mutant replication in HeLa cells at 60X magnification, showing vacuolar replication. Internalized 488-dextran was used to identify vacuolar** ***Salmonella.*** Left panel shows mCherry *Salmonella* artificially colored to reveal both low (blue/purple) and high (yellow/white) intensity bacteria. Right panels show an overlay of mCherry *Salmonella* (red) and Dextran-488 (green). Compare dextran positive vacuolar bacteria to dextran negative cytosolic bacteria. Times post infection are indicated.
(MOV)

Movie S5 **Live-cell imaging of** ***Salmonella*** **Typhimurium WT replication in HeLa cells at 60X magnification, showing cytosolic replication.** Internalized 488-dextran was used to identify vacuolar *Salmonella.* Left panel shows mCherry *Salmonella* artificially colored to reveal both low (blue/purple) and high (yellow/white) intensity bacteria. Right panels show an overlay of mCherry *Salmonella* (red) and Dextran-488 (green). Compare dextran positive vacuolar bacteria to dextran negative cytosolic bacteria. Times post infection are indicated.
(MOV)

Movie S6 **Live-cell imaging of** ***Salmonella*** **Typhimurium SPI2 mutant replication in HeLa cells at 60X magnification, showing cytosolic replication.** Internalized 488-dextran was used to identify vacuolar *Salmonella.* Left panel shows mCherry *Salmonella* artificially colored to reveal both low (blue/purple) and high (yellow/white) intensity bacteria. Right panels show an overlay of mCherry *Salmonella* (red) and Dextran-488 (green). Compare dextran positive vacuolar bacteria to dextran negative cytosolic bacteria. Times post infection are indicated.
(MOV)

Movie S7 **WT replication at 20X magnification over multiple fields of view.** Propidium iodide was used to identify HeLa cells with compromised plasma membranes. Each frame contains an overlay of GFP *Salmonella* (green), propidium iodide (red) and phase contrast (gray). Times post infection are indicated.
(MOV)

Movie S8 **SPI2 mutant replication at 20X magnification over multiple fields of view.** Propidium iodide was used to identify HeLa cells with compromised plasma membranes. Each frame contains an overlay of GFP *Salmonella* (green), propidium iodide (red) and phase contrast (gray). Times post infection are indicated.
(MOV)
